# Male partner involvement in antenatal care: implications for maternal health services in Juba, South Sudan

**DOI:** 10.1016/j.xagr.2026.100679

**Published:** 2026-07-27

**Authors:** Zechariah J. Malel, Ezbon WApary, Alnazir Osman Abdalla, Esraa Ahmed Mohammed, Yak Ring Tong Yak, Moses Mangar Aleth, Bok Phillip Nhial Bok, Mohammed Eissa Mohammed

**Affiliations:** 1 School of Medicine, University of Juba, Juba City, Republic of South Sudan (Malel, Wapary, Abdalla, Mohammed, Yak, Aleth, Bok, Mohammed); 2 Association of Gynecologists and Obstetricians of South Sudan (AGOSS), Juba City, Republic of South Sudan (Malel)

**Keywords:** antenatal care, male involvement, maternal healthcare

## Abstract

**BACKGROUND:**

Male partner involvement in antenatal care (ANC) is recognized as an important strategy for improving maternal and neonatal health outcomes. Active participation of male partners enhances support for pregnant women, promotes timely healthcare utilization, and improves adherence to ANC recommendations. Despite these benefits, male involvement in ANC remains low in many settings. This study assessed the level of male partner involvement, influencing factors, and partner knowledge regarding ANC services in Juba County, South Sudan.

**OBJECTIVE:**

To assess the level of male partner involvement in antenatal care and its association with maternal healthcare utilization among women attending maternal health services in Juba County, South Sudan.

**STUDY DESIGN:**

A facility-based cross-sectional study was conducted from November to December 2025 among women attending ANC services in the selected public health facilities in Juba County, South Sudan. Data were collected using structured questionnaires to assess the level of male partner involvement in ANC visits and factors influencing participation. Descriptive statistics and logistics analyses were performed, with statistical significance set at *P*<.05 to identify the associated factors.

**RESULTS:**

Overall, 50.4% (95% confidence intervals: 45.7–55.5) of women reported being accompanied by their male partners to at least one ANC visit. Significant factors influencing male involvement included women’s education level, partner’s age, partner’s education level, health worker encouragement, and a welcoming ANC environment (*P*<.05). The main barrier to involvement was work-related commitments (59.6%). Women whose partners had secondary level education and greater awareness of maternal health were more likely to report male participation in ANC visits.

**CONCLUSION:**

Male partner involvement in ANC remains inadequate, potentially limiting improvements in maternal and neonatal health outcomes. Educational attainment and supportive health facility practices were associated factors with male with participation. Interventions aimed at increasing male awareness, addressing cultural barriers, and creating male-friendly ANC services are needed to enhance participation and support safer pregnancies and childbirth.

**AJOG Global Reports at a Glance**

**Why was this study conducted?**

Male partner involvement in antenatal care is associated with improved maternal health outcomes, but evidence from South Sudan is limited.

**Key findings**

Our study revealed that 50% of pregnant women were accompanied by their male partner to at least one ANC visit. The study demonstrated that male involvement was associated with increased ANC attendance and utilization of skilled delivery services. Women’s education, partner education, partner age, health worker encouragement, and a male-friendly ANC environment were significantly associated with male involvement.

**What does this add to what is known?**

This study provides one of the first quantitative assessments of male partner involvement in ANC in South Sudan and highlights modifiable health system factors that could increase male participation in maternal healthcare.

## Introduction

Male partner involvement in maternal health has gained increasing recognition as a key strategy for improving maternal and newborn health outcomes. The International Conference on Population and Development (ICPD) held in Cairo in 1994 emphasized the importance of shared responsibility between men and women in reproductive health, encouraging men to actively participate in pregnancy, childbirth, and postnatal care.^1^ Subsequently, the World Health Organization (WHO) recommended male engagement as an important intervention for improving maternal and neonatal health outcomes and achieving the Sustainable Development Goals (SDGs) related to maternal and child survival.^2^

The partner involvement in antenatal care (ANC) is the active participation of male partners in maternal healthcare services. It extends beyond accompanying women to health facilities and includes participation in decision-making, emotional support, financial assistance, communication about pregnancy-related issues, birth preparedness, and support for healthcare utilization. Studies have shown that women whose partners are actively involved during pregnancy are more likely to attend ANC services, deliver in health facilities, utilize postnatal care services, and adhere to recommended maternal health interventions. Male engagement has also been associated with increased uptake of prevention of mother-to-child transmission (PMTCT) services, HIV testing, and improved maternal and newborn outcomes.^3^^,^^4^

Despite these benefits, male participation in ANC remains low in many low- and middle-income countries, particularly in sub-Saharan Africa (SSA).^5^ Cultural norms often view pregnancy and maternal healthcare as women’s responsibilities, limiting men’s involvement.^6^ Additional barriers include inadequate knowledge of maternal health, fear of HIV testing, work-related commitments, negative attitudes from healthcare providers, and health facilities that are not welcoming to men.^7^ Consequently, many women continue to make pregnancy-related healthcare decisions with limited support from their partners, contributing to delays in seeking and accessing maternal health services.^8^

The challenge of low male involvement is particularly relevant in South Sudan, which has one of the highest maternal mortality ratios in the world. Men often serve as primary decision-makers and controllers of household resources, giving them substantial influence over women’s access to maternal healthcare services. However, evidence suggests that male participation in ANC remains limited due to sociocultural beliefs, financial constraints, stigma, and weaknesses within the health system.^9^ In Juba County, barriers such as long waiting times, transportation costs, shortages of healthcare personnel, and perceptions that ANC services are exclusively for women may further discourage male engagement.

Although several studies from other African countries have documented the benefits and determinants of male involvement in ANC, there is limited quantitative evidence from South Sudan. Furthermore, variations in how male involvement is defined and measured have made comparisons across settings difficult. Understanding the level of male partner involvement and the factors’ influencing participation is essential for designing interventions that promote shared responsibility in maternal healthcare and improve maternal and neonatal outcomes.^10^

Therefore, this study aimed to assess the level of male partner involvement in antenatal care among women attending ANC services in Juba County, South Sudan, and to identify factors associated with their participation.

## Materials and methods

### Study design

A descriptive cross-sectional study was conducted to determine the prevalence of male partner involvement in ANC among pregnant women attending ANC services in selected public health facilities in Juba County, South Sudan.

### Study setting

The study was conducted in Juba County, Central Equatoria State, South Sudan. As the capital city of South Sudan, Juba has the largest and most diverse population in the country, comprising both urban and peri-urban communities, where men are commonly regarded as the primary household decision-makers and control financial resources, which substantially influence women’s access to maternal healthcare services. Despite this influential role, male attendance at ANC and other maternal health services remains relatively low due to prevailing cultural norms, work-related responsibilities, and perceptions that pregnancy and childbirth are primarily women’s responsibilities. Data were collected from 3 public health facilities providing ANC services: Juba Teaching Hospital, which is the main country tertiary national referral hospital, Juba Military Hospital which is the second tertiary hospital in city serving military personnel and civilians, and Munuki Primary Health Care Centre, the high volume primary healthcare facility providing comprehensive maternal and child health services. All 3 facilities are located in Juba.

### Sample size and sampling technique

The sample size was calculated using the single population proportion formula, with a prevalence of 51%, 95% confidence, and a 5% margin of error. After accounting for a 10% nonresponse rate, the final sample size was 427 individuals. To enroll eligible pregnant women who attend ANC clinics at the designated facilities, a randomized sample technique was used. The total sample size of 427 participants was proportionally allocated to the 3 study facilities based on their average monthly ANC attendance using the formula: n_i = \frac{N_i}{N} \times n

Where n_i is the sample size allocated to facility i, N-i is the average monthly ANC attendance at facility i, N is the total average monthly ANC attendance across all facilities, and n is the total sample size (427). Based on this approach, 205 participants (48.0%) were recruited from Juba Teaching Hospital, 102 (24.0%) from Juba Military Hospital, and 120 (28.0%) from Munuki Primary Health Care Centre. To reach the total sample size, 20 participants were randomly recruited daily across the 3 health facilities (6–7 participants each facility per day) over the course of 5 working days (Monday through Friday) until the desired sample size was met.

### Data collection tool and procedures

Data were collected using a structured questionnaire administered through interviewer-assisted and self-administered approaches for those who preferred to complete the questionnaire independently. It was developed by the research team and under-want face and content validity assessment by experts from the Department of Community Medicine, University of Juba, before data collection. The questionnaire captured information on socio-demographic characteristics, obstetric history, and male partner involvement in ANC. The tool was pretested among pregnant women attending antenatal care at a health facility not included in the main study. Reliability was assessed using Cronbach’s alpha for relevant scale items. Based on the pretest findings, minor revisions were made to improve the clarity and wording of selected questions before inception of data collection. Data collection was conducted between November 4 and December 4, 2025, using randomized study procedure by trained medical students under the supervision of the principal investigator. Eligible participants were identified at ANC clinics, informed about the study, and invited to participate after providing written informed consent. All interviews were conducted in a private setting within the health facility, away from partners, relatives, and other patients, to ensure confidentiality and minimize social desirability bias. Participants were interviewed individually, and privacy was maintained throughout the data collection process.

### Data analysis

Data were checked for completeness and consistency before being coded and entered into SPSS version 27 for analysis. Descriptive statistics, including frequencies, percentages, means, and standard deviations, were used to summarize participant characteristics and estimate the prevalence of male partner involvement in ANC with 95% confidence intervals (CI). Associations between male partner involvement and independent variables were assessed using chi-square tests and logistic regression analysis. Statistical significance was determined at a *P* value of less than .05.

### Ethical considerations

Ethical approval was obtained from the South Sudan’s National Ministry of Health Ethical Committee prior to data collection in accordance with Helinski Declaration. The approval reference number detailed MOH/RERB/A/10/2025. Written informed consent was obtained from all participants after explaining the study objectives, procedures, and their right to withdraw at any stage without consequences. Confidentiality was maintained by using anonymous codes instead of names, and the data were stored securely and accessible only to the research team. Participants aged below 18 years who were legally married and attending antenatal care were considered emancipated minors in accordance with the national ethical guidelines and were therefore eligible to provide informed consent independently. For participants below 18 years who were not emancipated, written parental/guardian consent was obtained in addition to participant assent before enrolment.

## Results

### Demographic characteristics of women attending ANC in public health facilities in Juba County, South Sudan

Table 1 outlines the socio-demographic characteristics of 427 women attending ANC in Juba County. The majority are young adults, primarily aged 20 to 25 years (38.4%). Educationally, 29.5% have no formal education, and most women are unemployed (68.1%). Marital status shows 76.6% are in monogamous marriages, with partners mostly aged 26 to 30 years. Partners generally have higher educational attainment, with 45% having tertiary education, and are more economically active (36.5% formally employed).

**Table 1:** Demographic characteristics of women attending ANC in public health facilities in Juba County, South Sudan

	*n*=427	% (95% CI)
Participant’s current age (y)		
15–19	53	12.4 (9.4–15.7)
20–25	164	38.4 (34–43.1)
26–30	150	35.1 (30.7–39.8)
31–35	36	8.4 (5.9–11)
36–40	21	4.9 (2.8–7.3)
41–45	3	0.7 (0–1.6)
Current participant’s highest level of education		
No formal education	126	29.5 (25.1–34)
Primary education	121	28.3 (24.4–33.3)
Secondary education	113	26.5 (22.2–30.7)
Tertiary/higher education	67	15.7 (12.4–19.2)
Main occupation/source of income		
Unemployed/housewife	291	68.1 (63.5–72.6)
Casual laborer/farmer	7	1.6 (0.7–3)
Employed (public/private sector)	30	7 (4.7–9.6)
Own business/self-employed	44	10.3 (7.3–13.1)
Student	55	12.9 (9.8–15.9)
Current marital status		
Married/living with partner (monogamous)	327	76.6 (72.6–81)
Married/living with partner (polygamous)	82	19.2 (15.5–23)
Separated/divorced/widowed	18	4.2 (2.3–6.3)
Partner’s current age (in y)		
20–25	31	7.3 (4.9–9.8)
26–30	142	33.3 (28.8–37.7)
31–35	93	21.8 (18–26)
36–40	75	17.6 (13.8–21.3)
41–45	66	15.5 (11.7–19)
46 above	20	4.7 (2.8–6.8)
Partner’s highest level of education		
No formal education	67	15.7 (12.2–19.4)
Primary education	69	16.2 (12.4–19.7)
Secondary education	99	23.2 (19.2–27.2)
Tertiary/higher education	192	45 (40.5–49.4)
Partner’s main occupation/source of income		
Unemployed	96	22.5 (18.7–26.7)
Casual laborer/farmer	25	5.9 (3.7–8.2)
Employed (public/private sector)	156	36.5 (32.1–41.2)
Own business/self-employed	150	35.1 (30.7–39.3)

Table 2 summarizes key reproductive metrics among women attending ANC in Juba County. The mean gravidity was 3.43 pregnancies (SD: 2.05), indicating substantial variability among participants. The mean parity was 2.62 living children (SD: 2.01), suggesting some pregnancies ended in miscarriage or loss. The mean gestational age at ANC attendance was 4.83 months (SD: 2.16), indicating that many women attend during the second trimester.

**Table 2:** Obstetrics characteristics of women attending ANC in public health facilities in Juba County, South Sudan

			95% CI	
	*n*=427		LL	UL
Number of pregnancies (gravida), including this current one				
	Mean	3.43	3.25	3.62
	Std. dev	2.05	1.93	2.17
Number of deliveries (parity)				
	Mean	2.62	2.44	2.81
	Std. dev	2.01	1.86	2.14
Gestational age of the current pregnancy by months				
	Mean	4.83	4.63	5.05
	Std. dev	2.16	2.03	2.29

### Antenatal care attendance, male partner involvement during ANC visits, and reasons for nonaccompaniment among pregnant women attending public health facilities in Juba County, South Sudan

Table 3 presents ANC attendance patterns, male partner involvement during ANC visits, and reasons for nonaccompaniment. The study revealed that in ANC attendance in Juba County, South Sudan, 41.7% of women attended one visit. Overall, 78.5% (95% CI: 74.5–82.4) of women had attended fewer than 3 ANC visits during the current pregnancy, while 21.5% (95% CI: 17.6–25.5) had attended 3 or more visits. Male partners accompanied 50.4% (95% CI: 45.7–55.5) of women to at least one ANC visit. Among those accompanied, 36.9% reported that their partner attended once, whereas 29.6% reported 4 or more accompanying visits. More than half (57.3%) indicated that their partner remained throughout the entire consultation.

**Table 3:** Antenatal care attendance, male partner involvement during ANC visits, and reasons for nonaccompaniment among pregnant women attending public health facilities in Juba County, South Sudan (*n*=427)

	*n*=427	% (95%CI)
Total number of ANC visits in the current pregnancy, including this last visit		
0	6	1.4 (0.5–2.6)
1	178	41.7 (37.2–46.4)
2	113	26.5 (22.2–30.9)
3	38	8.9 (6.3–11.7)
4	37	8.7 (6.3–11.7)
5	32	7.5 (4.9–10.1)
6	8	1.9 (0.7–3.3)
8	15	3.5 (1.9–5.4)
Overall number of ANC visits		
<3	335	78.5 (74.5–82.4)
>3	92	21.5 (17.6–25.5)
Male partner accompaniment for any ANC visit during this current pregnancy		
Yes	215	50.4 (45.7–55.5)
No	212	49.6 (44.5–54.3)
Frequency of the male partner accompaniment for ANC visits in this current pregnancy		
Once	75	36.9 (29.6–43.8)
Twice	59	29.1 (23.2–35.5)
Three times	9	4.4 (2–7.9)
Four or more times	60	29.6 (23.2–36)
Male partner stayed for the whole duration of the visit, or only drop off/pick up		
Yes,stayed with her for the whole visit duration	129	57.3 (51.1–63.6)
Only dropped off or picked up her from ANC	25	11.1 (7.1–15.6)
Both	71	31.6 (25.8–37.3)
Reasons for nonaccompaniment by the partners to the ANC		
Partner was too busy with work/searching for income	229	59.6 (54.7–64.1)
Partner believed it is only a woman’s responsibility	16	4.2 (2.3–6.3)
Lack of transport/long distance to the facility	52	13.5 (10.4–16.9)
Fear of being tested for HIV/AIDS	2	0.5 (0–1.3)
Partner did not know he was expected to attend	2	0.5 (0–1.3)
Partner was away/not living together	6	1.6 (0.5–3.1)
Other	77	20.1 (16.4–24)

Among women whose partners did not accompany them, the most commonly reported reason was that the partner was busy with work or income-generating activities (59.6%), followed by other reasons (20.1%) and lack of transport or long distance to the health facility (13.5%). Fewer respondents cited beliefs that ANC is solely a woman’s responsibility (4.2%), partners living away (1.6%), fear of HIV testing (0.5%), or lack of awareness that men were expected to attend ANC (0.5%).

### Determining factors associated with male partner involvement in ANC visits in public health facilities in Juba County, South Sudan

Table 4 explores the factors influencing male partner accompaniment to ANC visits. Overall, 50.4% of male partners accompanied women to at least one ANC visit. Significant associations were found between male involvement and women’s education (*P*<.001, Phi=0.228) and partners’ age (*P*=.001, Phi=0.221) and education (*P*<.001, Phi=0.303). The more educated the woman or partner, the higher the likelihood of accompaniment. Notable health system factors were encouragement by health workers (*P*<.001, Phi=0.289) and the welcoming nature of the ANC environment (*P*=.001, Phi=0.187). In contrast, women’s age, occupation, marital status, partner’s occupation, and decision-making factors did not show significant association.

**Table 4:** Determining factors associated with male partner involvement in ANC visits in public health facilities in Juba County, South Sudan

Factors	Male partner accompaniment for any ANC visit during the current pregnancy					Total, *n* (%)			Chi-square			*P* value	Effect size (Phi/Cramér’s *V*)
	Yes, *n* (%)	No, *n* (%)											
Participant age (y)									10.704			.058	0.158
15–19	29 (54.7)	24 (45.3)				53 (12.4)							
20–25	79 (48.2)	85 (51.8)				164 (38.4)							
26–30	84 (56)	66 (44)				150 (35.1)							
31–35	10 (27.8)	26 (72.2)				36 (8.4)							
36–40	12 (57.1)	9 (42.9)				21 (4.9)							
41–45	1 (33.3)	2 (66.7)				3 (0.7)							
Participant highest level of education									22.222			<.001	0.228
No formal education	54 (42.9)	72 (57.1)				126 (29.5)							
Primary education	51 (42.1)	70 (57.9)				121 (28.3)							
Secondary education	60 (53.1)	53 (46.9)				113 (26.5)							
Tertiary/higher Education	50 (74.6)	17 (25.4)				67 (15.7)							
Main occupation/source of income									8.593			.072	0.142
Unemployed/house wife	143 (49.1)	148 (50.9)				291 (68.1)							
Casual laborer/farmer	5 (71.4)	2 (28.6)				7 (1.6)							
Employed (public/private sector)	13 (43.3)	17 (56.7)				30 (7)							
Own business/self-employed	18 (40.9)	26 (59.1)				44 (10.3)							
Student	36 (65.5)	19 (34.5)				55 (12.9)							
Current marital status										4.618		.099	0.104
Married/living with partner (monogamous)	156 (47.7)	171 (52.3)				327 (76.6)							
Married/living with partner (polygamous)	50 (61)	32 (39)				82 (19.2)							
Separated/divorced/widowed	9 (50)	9 (50)				18 (4.2)							
Partner’s current age (in y)										20.838		.001	0.221
20–25	23 (74.2)	8 (25.8)				31 (7.3)							
26–30	63 (44.4)	79 (55.6)				142 (33.3)							
31–35	60 (64.5)	33 (35.5)				93 (21.8)							
36–40	32 (42.7)	43 (57.3)				75 (17.6)							
41–45	30 (45.5)	36 (54.5)				66 (15.5)							
46 above	7 (35)	13 (65)				20 (4.7)							
Partner’s highest level of education										39.322		<.001	0.303
No formal education	25 (37.3)	42 (62.7)				67 (15.7)							
Primary education	18 (26.1)	51 (73.9)				69 (16.2)							
Secondary education	46 (46.5)	53 (53.5)				99 (23.2)							
Tertiary/higher education	126 (65.6)	66 (34.4)				192 (45)							
Partner’s main occupation/source of income										3.069		.381	0.085
Unemployed	50 (52.1)	46 (47.9)				96 (22.5)							
Casual laborer/farmer	10 (40)	15 (60)				25 (5.9)							
Employed (public/private sector)	85 (54.5)	71 (45.5)				156 (36.5)							
Own business/self-employed	70 (46.7)	80 (53.3)				150 (35.1)							
Number of ANC visit													
≤3	170 (50.7)	165 (49.3)				335 (78.5)							
>3	45 (48.9)	47 (51.1)				92 (21.5)							
Partner financial support for ANC-related costs (eg, clinic fees, medication, and transport) for this current pregnancy											2.633	.268	0.079
Yes, all costs				168 (49.6)			171 (50.4)	339 (79.8)					
Yes, some costs				24 (61.5)			15 (38.5)	39 (9.2)					
No				21 (44.7)			26 (55.3)	47 (11.1)					
Decision for ANC visit											6.781	.079	0.126
Woman decided alone				50 (49)			52 (51)	102 (23.9)					
Partner decided alone				13 (32.5)			27 (67.5)	40 (9.4)					
Joint decision between the couple			138 (54.1)				117 (45.9)	255 (59.7)					
Health worker recommendation/other family member			14 (46.7)				16 (53.3)	30 (7)					
Discussion with partner on the importance of ANC, safe delivery, or danger signs in pregnancy											0.103	.95	0.016
Yes, often discuss			134 (50.2)				133 (49.8)	267 (62.8)					
Yes, sometimes discuss			49 (50.5)				48 (49.5)	97 (22.8)					
No, never discuss			32 (52.5)				29 (47.5)	61 (14.4)					
Partner encouragement for adequate nutrition during this pregnancy											0.163	.922	0.02
Yes, always			182 (50.6)				178 (49.4)	360 (84.3)					
Yes, sometimes			18 (47.4)				20 (52.6)	38 (8.9)					
No, rarely or never			15 (51.7)				14 (48.3)	29 (6.8)					
Health workers encouragement for male partners to attend ANC along their pregnant women											35.58	<.001	0.289
Yes, they strongly encourage			190 (53.2)				167 (46.8)	357 (83.6)					
Yes, they sometimes encourage			24 (66.7)				12 (33.3)	36 (8.4)					
No, rarely or never encourage			1 (2.9)				33 (97.1)	34 (8)					
ANC environment (eg, waiting area, consultation room) welcoming to presence of male partners											14.884	.001	0.187
Yes, very welcoming			191 (51.5)		180 (48.5)			371 (86.9)					
Neutral/okay			22 (61.1)		14 (38.9)			36 (8.4)					
No, not welcoming at all			2 (10)		18 (90)			20 (4.7)					
Total			215 (50.4)		212 (49.6)			427 (100)					

Table 5 presents the multivariable logistic regression analysis of factors associated with male partner involvement in ANC visits. Significant predictors include women’s educational levels; those with secondary education (OR=8.321, *P*<.001), while primary education (OR=3.056, *P*=.058) was not significantly associated with partner involvement. Compared with the reference category, partners aged 26 to 30 years and 36 to 40 years had lower odds of accompanying their partners to antenatal care, as indicated by their respective odds ratios. Men’s education significantly affected involvement, with primary (OR=4.309, *P*=.001) and secondary (OR=5.992, *P*<.001) education correlating positively, while tertiary education was not significant. Compared with women who reported receiving strong encouragement from health workers, those who reported that male involvement was only sometimes encouraged or never encouraged had substantially lower odds of male partner accompaniment, corresponding to approximately 97.2% and 98.8% reductions in the odds, respectively. A welcoming ANC environment is also vital; neutrality (OR=0.034, *P*<.001) or lack of friendliness significantly decreases participation. Gravidity increases involvement (OR=2.008, *P*<.001), while having more living children decreases it (OR=0.432, *P*<.001). Overall, health system factors, education levels, gravidity, and parity are factors associated with male involvement, indicating the need for male-friendly services and community education to enhance participation in maternal health services in Juba County.

**Table 5:** Logistic regression model: determining factors associated with male partner involvement in ANC visits in public health facilities in Juba County, South Sudan

Predictor	*B*	SE	Wald	df	*P* value	OR	95% CI (LL–UL)	
Participant’s highest level of education								
No formal education^a^								
Primary education	1.117	0.589	3.6	1	.058	3.056	0.964	9.689
Secondary education	2.119	0.547	15.004	1	<.001	8.321	2.848	24.309
Tertiary/higher education	1.807	0.536	11.352	1	.001	6.091	2.129	17.426
Partner’s current age (in y)								
20–25^a^								
26–30	−2.038	0.736	7.663	1	.006	0.13	0.031	0.551
31–35	−0.552	0.603	0.837	1	.36	0.576	0.177	1.878
36–40	−1.505	0.638	5.556	1	.018	0.222	0.064	0.776
41–45	−0.166	0.628	0.069	1	.792	0.847	0.247	2.902
46 above	−0.618	0.653	0.894	1	.344	0.539	0.15	1.94
Partner highest level of education								
No formal education^a^								
Primary education	1.461	0.425	11.786	1	.001	4.309	1.872	9.921
Secondary education	1.79	0.431	17.29	1	<.001	5.992	2.577	13.936
Tertiary/higher education	0.53	0.315	2.831	1	.092	1.699	0.916	3.148
Health workers encouragement for male partners to attend ANC along their pregnant women								
Yes, they strongly encourage^a^								
Yes, they sometimes encourage	−3.588	1.19	9.091	1	.003	0.028	0.003	0.285
No, rarely or never encourage	−4.388	1.28	11.759	1	.001	0.012	0.001	0.153
ANC environment (eg, waiting area, consultation room) welcoming to presence of male partners								
Yes, very welcoming^a^								
Neutral/okay	−3.37	0.922	13.347	1	<.001	0.034	0.006	0.21
No, not welcoming at all	−2.537	1.002	6.411	1	.011	0.079	0.011	0.564
Number of pregnancies (gravida), including this current one	0.697	0.192	13.126	1	<.001	2.008	1.377	2.928
Number of deliveries including the current pregnancy (parity)	−0.84	0.185	20.558	1	<.001	0.432	0.3	0.621
Current months of pregnancy	0.108	0.066	2.655	1	.103	1.114	0.978	1.268

a Reference category.

### Knowledge, communication, and supportive practices related to male partner involvement in antenatal care services in public health facilities in Juba County, South Sudan

Table 6 provides insights into male partner engagement in ANC in Juba County, South Sudan. Key findings include that 62.5% of men frequently discuss ANC, safe delivery, and danger signs with their partners, while 14.8% do not engage in these discussions, highlighting knowledge gaps in some households. Regarding nutritional support during pregnancy, 84.3% of male partners ensure adequate nutrition, indicating a strong awareness of maternal dietary needs. Health workers also play a vital role; 83.6% actively encourage male attendance at ANC, facilitating their involvement and support. Moreover, 82.7% of health workers include male partners in consultations, which promotes inclusive communication and knowledge acquisition. Overall, the data reflect a generally positive attitude toward male involvement in maternal health, while also pointing out that a minority remain disengaged.

**Table 6:** Knowledge, communication, and supportive practices related to male partner involvement in antenatal care services in public health facilities in Juba County, South Sudan (*n*=427)

	*n*=427	% (95% CI)
Discussion with the partner on the importance of ANC, safe delivery, or danger signs in pregnancy		
Yes, often discussed	267	62.5 (58.1–67.2)
Yes, sometimes discuss	97	22.7 (18.5–26.7)
No, never discuss	63	14.8 (11.2–18.5)
Partner support to ensure adequate nutrition during the current pregnancy		
Yes, always	360	84.3 (80.8–87.8)
Yes, sometimes	38	8.9 (6.3–11.5)
No, rarely or never	29	6.8 (4.4–9.4)
Health workers encouragement for male partners to attend ANC along their pregnant women		
Yes, they strongly encourage	357	83.6 (80.1–87.1)
Yes, they sometimes encourage	36	8.4 (5.9–11.5)
No, rarely or never encourage	34	8 (5.6–10.5)
Provision of consultation to both couple by the health workers during partner accompaniment to ANC		
Yes, always	353	82.7 (78.9–86.2)
Yes, sometimes	46	10.8 (7.7–13.8)
No, only for pregnant women	22	5.2 (3–7.5)
No, partner didn’t show up in the ANC	6	1.4 (0.5–2.6)

## Discussion

Male partner involvement in ANC is increasingly recognized as a critical component of maternal and newborn health.^11^ This study assessed the level of male partner involvement, associated factors, and partner knowledge regarding ANC services among women attending public health facilities in Juba County, South Sudan. The findings demonstrate that approximately half (50.4%) of women reported being accompanied by their male partners to at least one ANC visit during the current pregnancy. Although this level of involvement is encouraging compared to reports from several low-income settings, it remains insufficient to achieve the full benefits of male engagement in maternal healthcare.^12^

The prevalence of male partner accompaniment observed in this study is comparable to findings from studies conducted in other sub-Saharan African countries, where male involvement in ANC ranges between 30% and 60%.^13^ The moderate level of participation may reflect increasing awareness of the importance of maternal health services and the efforts of health facilities to encourage male attendance.^14^ Nevertheless, nearly half of women attended ANC without partner accompaniment, indicating persistent barriers to male participation. Given the influential role men play in household decision-making and resource allocation in South Sudan, limited involvement may negatively affect timely healthcare-seeking, birth preparedness, and adherence to maternal health recommendations.^15^

Women’s educational attainment was a significant predictor of male partner involvement. Women with secondary and tertiary education were substantially more likely to be accompanied by their partners compared with women who had no formal education. Similar findings have been reported in studies from Ethiopia, Uganda, and Kenya, where education improves awareness of maternal health services, communication between couples, and healthcare utilization. Educated women may be more empowered to encourage partner participation and to recognize the benefits of shared responsibility during pregnancy.^16^^,^^17^ This finding underscores the broader importance of female education in improving maternal health outcomes.

Partner education was also strongly associated with ANC involvement. Men with secondary education were significantly more likely to participate in ANC compared with those without formal or with only primary education. Education may enhance understanding of pregnancy-related risks and the value of preventive healthcare services, thereby promoting supportive behaviors. Interestingly, tertiary education was not independently associated with involvement after adjustment for other factors, suggesting that factors beyond formal education, such as work commitments and competing responsibilities, may influence participation among highly educated men.^12^

The study further revealed that younger male partners were more likely to participate in ANC than older partners. Men aged 26 to 30 years and 36 to 40 years were significantly less likely to accompany their partners compared with those aged 20 to 25 years. This finding may reflect generational differences in attitudes toward gender roles and maternal healthcare.^18^ Younger men may be more receptive to contemporary messages promoting shared responsibility in reproductive health, whereas older men may adhere more strongly to traditional norms that regard pregnancy and ANC as women’s responsibilities.^19^

Health system factors emerged as some of the strongest determinants of male involvement. Women who reported that health workers actively encouraged male attendance were significantly more likely to be accompanied by their partners. Similarly, perceptions of a welcoming ANC environment were strongly associated with male participation. These findings highlight the critical role healthcare facilities play in promoting male engagement. Previous studies have shown that male-friendly services, including couple-focused health education, flexible clinic hours, and inclusive consultation practices, can substantially increase male attendance.^20^ The current findings suggest that strengthening provider attitudes and creating supportive environments may be among the most effective interventions for improving male involvement in South Sudan.

An important finding was that work-related commitments represented the most common barrier to male participation, accounting for nearly 60% of reported reasons for nonattendance. Similar challenges have been documented throughout sub-Saharan Africa, where men often prioritize income-generating activities to support their families.^21^ In resource-constrained settings such as South Sudan, missing work to attend ANC may carry significant economic consequences. This suggests that interventions aiming to improve male involvement should consider more flexible appointment schedules, weekend clinics, workplace sensitization programs, and community-based approaches that accommodate men’s work responsibilities.

The relationship between reproductive history and male involvement was also noteworthy. Increasing gravidity was associated with higher odds of male participation, while higher parity reduced the likelihood of involvement. Men may become more engaged as couples gain experience with pregnancy and childbirth, particularly if previous pregnancies involved complications.^22^ Conversely, couples with several living children may perceive pregnancy as routine and therefore devote less attention to ANC attendance.^23^ This finding highlights the need for continued education emphasizing the importance of ANC during every pregnancy, regardless of previous childbirth experiences.

The assessment on the communication with the male partner on ANC revealed generally positive levels of awareness and engagement. More than three-fifths of women reported frequent discussions with their partners regarding ANC, safe delivery, and pregnancy danger signs, while the vast majority indicated receiving nutritional support during pregnancy. Additionally, most respondents reported that health workers encouraged male attendance and involved partners during consultations. These findings suggest that when men engage with maternal health services, they can contribute meaningfully to pregnancy support and decision-making. However, the proportion of couples who never discussed maternal health issues indicates that knowledge gaps and communication barriers remain among some households.^24^ Healthcare workers’ encouragement was significantly associated with increased male partner involvement in antenatal care. This finding suggests that healthcare providers play an important role in promoting male participation by encouraging couples to attend ANC together, providing health education, and creating a welcoming environment for men. Similar findings have been reported in other sub-Saharan African studies, highlighting that provider support can improve male engagement in maternal healthcare and contribute to better maternal and newborn health outcomes.

The findings of this study have important implications for maternal health programs in South Sudan. Interventions aimed at increasing male involvement should prioritize community education, couple-centered health promotion, and strategies that address sociocultural norms limiting male participation. Health facilities should continue strengthening male-friendly ANC services by encouraging partner attendance, providing inclusive counselling, and reducing structural barriers. Engaging community leaders, religious leaders, and men’s groups may further help transform social norms and promote shared responsibility for maternal and newborn health.

This study has several limitations. The cross-sectional design limits the ability to establish causal relationships between identified factors and male involvement. Data were based on self-reports from women, which may be subject to recall and social desirability bias.^25^ Furthermore, the study was conducted in public health facilities within Juba County and may not fully represent rural populations or women who do not attend ANC services. Despite these limitations, the study provides valuable evidence on male partner involvement in ANC in South Sudan, where such data remain limited.

Overall, the study demonstrates that male partner involvement in ANC in Juba County remains moderate and is influenced by educational, demographic, and health system factors. Strengthening male-friendly health services, enhancing community awareness, and addressing structural barriers to participation could substantially improve male engagement and contribute to better maternal and neonatal health outcomes in South Sudan.

## Conclusion

Male partner involvement in ANC in Juba County was moderate, with only half of women reporting partner accompaniment. Education, health worker encouragement, and a welcoming facility environment were key factors associated with involvement, while work commitments were the main barrier. Strengthening male-friendly ANC services and community awareness may improve male participation and maternal health outcomes.

## CRediT authorship contribution statement

**Zechariah J. Malel:** Writing – original draft, Supervision, Conceptualization. **Ezbon WApary:** Writing – review & editing, Formal analysis. **Alnazir Osman Abdalla:** Writing – review & editing, Investigation. **Esraa Ahmed Mohammed:** Writing – review & editing, Investigation. **Yak Ring Tong Yak:** Writing – review & editing, Investigation. **Moses Mangar Aleth:** Writing – review & editing, Investigation. **Bok Phillip Nhial Bok:** Writing – review & editing, Investigation. **Mohammed Eissa Mohammed:** Writing – review & editing, Investigation.
